# Autism spectrum disorders and atopic dermatitis: a new perspective from country-based prevalence data

**DOI:** 10.1186/s12948-021-00166-5

**Published:** 2021-12-20

**Authors:** Alessandro Tonacci, Giovanni Pioggia, Sebastiano Gangemi

**Affiliations:** 1 Clinical Physiology Institute, National Research Council of Italy (IFC-CNR), Via Moruzzi 1, 56124 Pisa, Italy; 2 Institute for Biomedical Research and Innovation, National Research Council of Italy (IRIB-CNR), 98164 Messina, Italy; 3 Department of Clinical and Experimental Medicine, School and Operative Unit of Allergy and Clinical Immunology, University of Messina, 98125 Messina, Italy

**Keywords:** Atopic dermatitis, Atopy, Autism spectrum disorders, Immune system, Neurodevelopmental disorders

## Abstract

Recently, a continuous growth for both neurodevelopmental and atopic diseases’ incidence was seen throughout the world. Notably, autism spectrum disorders (ASD) and atopic dermatitis (AD) attracted the attention of clinicians and scientists for their impact on the quality of life of patients, starting during childhood. Despite a number of hypotheses about common pathogenesis between the two disorders, uncertainty is still present, and data coming from various parts of the world are contradictory. Fortunately, works recently published have brought useful material for comparative analysis to the benefit of the scientific community, making large scale, country-based perspectives methodologically viable. In light of that, the present study took into account uniform data, available from country-based registries or related publications, dealing with the prevalence of the two conditions around the world, and tried to setup a simple correlation analysis between the two. According to such data, the growth of AD and ASD prevalence appear uncorrelated, leading to hypothesise that, if a common etiopathological pathway exists between the two conditions, they are likely to interact to each other due to a complex interplay of co-morbidities, genes and environmental players not enough explained by a simple correlation analysis. Such facts are worth investigation in future research.

**Supplementary Information:**

The online version contains supplementary material available at 10.1186/s12948-021-00166-5.

## Introduction

Atopic diseases are continuously growing in terms of worldwide prevalence even in infants and children, starting very early during infancy with atopic dermatitis (AD), followed by allergic rhinitis and asthma later in life [1], in the clinical pipeline known as the “atopic march” [2]. Their cause is not fully revealed, yet, but their increased global prevalence, particularly concerning AD, is not to be due to genetics alone, but can be attributable to an interplay with evolving environmental exposures, at least in predisposed individuals [3].

Notably, environmental agents, including pollutants and allergens, are associated with an increasing prevalence of the disease. Other environmental players, such as tobacco exposure, microbes, diet, nutrients, together with genetic variants, including filaggrin mutations, could lead to the deficiency of the skin barrier and the immune system, overall, paving the way for AD onset already early during life [4].

Quite surprisingly, atopic disorders [5], and in particular AD, were found to be somewhat associated with a number of psychiatric conditions, also starting during childhood [6, 7], among which autism spectrum disorders (ASD) are probably the best known and most prevalent worldwide.

Somewhat similarly to AD, also ASD pathogenesis is still unclear, but also in this case mounting evidence supports the hypothesis for a significant gene-environment interaction in its etiology [8].

A recent study on this topic attempted at making clarity based on population data from 5 countries (Denmark, Finland, Israel, Sweden and Western Australia). The authors found the heritability of ASD to be around 80%, with the variation in ASD occurrence mostly due to inherited genetic influences, and no evidence for related maternal effects [9]. Interestingly, the prevalence of AD in children living in Western countries is rising in a similar manner when compared to ASD, with hypothesised bidirectional association between them, suggesting a shared pathogenesis underlying the two, apparently far, conditions [1]. Several hypotheses were raised about a possible common pathway between the two disorders, including the dysregulation of common microRNAs, such as miR-146 and miR-155 [10], the co-existence of higher concentrations of pro-inflammatory cytokines in both atopic and neuropsychiatric disorders [11], or the action of overexpressed inflammatory mediators released during atopic responses that could have affected neural circuitry in genetically susceptible children [12].

More specifically, genetic variants in Stat6, pivotal element in the regulation of Th2 immune response, are known to be associated with atopy. But Stat6 is also found to be largely expressed in the central nervous system and to play a key role in the pathogenesis of some neuropsychiatric conditions, including ADHD, in turn largely co-morbid to ASD [6]. This, still indirect, possible association has further fostered the hypothesis for a somewhat connection between atopy and neuropsychiatric, or neurodevelopmental disorders.

Nevertheless, in this complex and quite puzzled framework, two of the largest population-based longitudinal studies published to date hypothesised a role for AD as an early precursor for ASD [13, 14]. According to the authors, toddlers affected from AD as young as 3 years old are at higher risk of developing ASD or ADHD later during childhood, especially in presence of other atopic comorbidities such as allergic rhinitis, allergic conjunctivitis, and asthma. Lee et al. [13] hypothesised a role for AD as a trigger for an immunological cascade comprising mast cells activation, in turn inducing the release of pro-inflammatory cytokines, mostly IL-6, whose levels were seen to be elevated in ASD patients and to modulate the autistic-like behaviour in animal models [15]. Such elevated cytokine levels found in ASD patients can bring to damage to the blood–brain barrier, in turn playing a key role in the etiopathogenesis of ASD and other neuropsychiatric conditions.

Both AD and ASD are actually under the magnifying glass of clinical research in the respective medical fields (allergology/immunology and pediatric neuropsychiatry, respectively) due to their increasing prevalence and to the attention of physicians and pharmaceutical industry. However, despite this fact, until a few years ago, the scientific literature experienced a lack of uniform correlation analyses between their respective prevalence in a significant number of nations from all over the world, except some isolated attempts to follow the prevalence trend of the two conditions longitudinally in a given country [16–18]. This, apparently strange phenomenon, was possibly due to a lack of standardization in clinical criteria that might have prevented researchers from carrying out such analysis in a reliable fashion.

Fortunately, in more recent times, larger databases have been published, therefore allowing for more exhaustive comparisons between the data pertaining the two conditions. Indeed, a big amount of countries are now included within uniform data collection reported online (https://worldpopulationreview.com/country-rankings/autism-rates-by-country) or in scientific literature [19] for both conditions, making the analysis less complicated than before.

## Materials and methods

In light of the still existing literature gap, and taking advantage of the novel, free data availability, we sought to correlate the information retrieved from [19] and https://worldpopulationreview.com/country-rankings/autism-rates-by-country between them, consisting of prevalence data from 186 countries dealing with AD and ASD prevalence worldwide. Statistical analysis was conducted using SPSS v.23 (IBM Corp., Armonk, NY, USA).

The distribution of the available data has driven us to adopt a non-parametric approach for correlations, using the two-tailed Spearman’s Test to verify the common trends for the two variables.

## Results and discussion

**Fig. 1:**
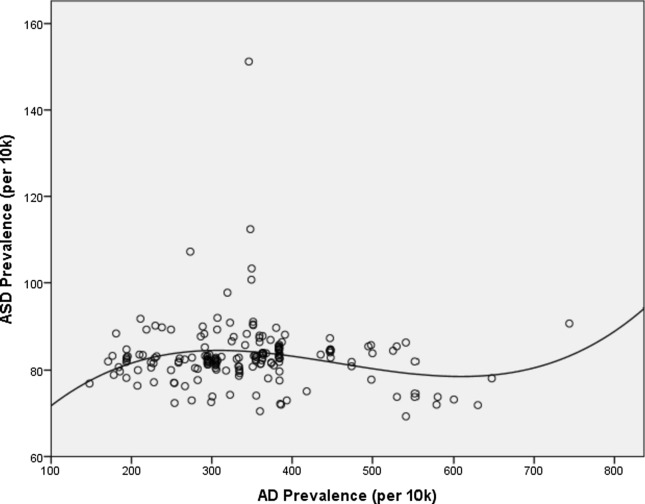
Regression curve, with cubic fit, between AD and ASD prevalence in the 186 countries whose data are available (complete data available as Additional file 1)

As expected, possibly due to the hypothesised complexity in the interaction between those two conditions, statistical significance was not reached (r  = 0.061, p  = 0.412), but at the same time interesting observations can be derived. In fact, taking AD prevalence as the independent variable and, consequently, ASD prevalence as dependent, and attempting to fit the two variables compared to each other, the best fit occurred applying a cubic fit, with r^2^  = 0.049 and p  = 0.026. This leads to the hypothesis that a somewhat relationship between the two prevalence datasets could exist, even if its magnitude can be deemed as quite reduced. Figure 1 displays the resulting fit.

As displayed, except for some outliers, the growth of ASD prevalence changes together with that of AD prevalence according to a cubic trend, possibly leading to consider the existence of a complex interplay between genes and environment in both AD and ASD, possibly suggesting a somewhat common etiopathological pathway between the two, disorders.

The main outliers are all represented by countries situated in the Middle East (Qatar, United Arab Emirates, Oman, Bahrain, Saudi Arabia), where ASD prevalence rates are surprisingly higher, without a similarity in any corresponding AD rise. This fact could be due to a number of external factors, possibly affecting the two conditions in a different manner. One of the possible, more reasonable explanations, is that Middle East countries have been experiencing a significant deficit in terms of serum Vitamin D levels, with several recommendations to undergo their supplementation already in women during pregnancy to prevent from bone diseases, immune malfunctioning and nervous system disturbances [20]. As such, hypovitaminosis D was associated with a higher risk of ASD pathogenesis [21], with convincing etiopathological explanations [22]. In addition, children with lower serum levels of Vitamin D appear to improve their clinical status after Vitamin D supplementation, with a significant reduction of psychiatric manifestations [23].

In addition, also AD severity was seen to be correlated with low levels of Vitamin D, and recent studies performed on infants confirmed this evidence [24, 25]. A possible role for Vitamin D in terms of symptoms improvements related to AD was suggested, with the optimal dosage of such compound seen to support a correct disease management and an improved clinical course [25, 26]. Such result is probably due to the fact that children with Vitamin D deficiency experience a weakness of the immune system, with lower levels of NKT lymphocytes and T-regulatory lymphocytes, and higher concentrations of interleukin-22, leading to the hypothesis for a proinflammatory alert state and for a significant weakness of the immune system in such individuals [25].

## Conclusions

Under such premises, our study is intended to stimulate future studies on this topic. Such researches should require: (i) a correlation of wider amount of epidemiological data for AD and ASD, but also referring to other immune, as well as neurodevelopmental disorders, and to environmental data, as desirable and where possible; (ii) a deeper investigation about the etiopathological pathways linking the two conditions to explain possible overlapping and to hypothesise innovative treatments for both disorders.

Such future steps are mandatory to confirm the existence of a clear relationship between the etiopathology of AD and ASD and to tailor new investigations about such link in a more focused manner to address the gaps still existing in the scientific literature. Also, this would ultimately lead to a broader understanding of the overall framework involving atopy and neurodevelopmental disorders, their causality and, hopefully, innovative and reliable strategies for challenging such conditions already during toddlerhood and early childhood.

## Supplementary Information

**Additional file 1: Table S1.** Details about prevalence of ASD and AD in the retrieved countries.

## Data Availability

The datasets generated and/or analysed during the current study are available in the repositories mentioned within the references [1, 3–5].
