# Supplementary material for: Autism spectrum disorders and atopic dermatitis: a new perspective from country-based prevalence data
Source: Clin Mol Allergy. 2021 Dec 20;19:27. doi: 10.1186/s12948-021-00166-5 (PMC8691085; doi:10.1186/s12948-021-00166-5)
Supplement: Supplementary file 1 — Additional file 1: Table S1. Details about prevalence of ASD and AD in the retrieved countries. [file 12948_2021_166_MOESM1_ESM.docx]

Supplementary Material

Suppl. Table 1: Details about prevalence of ASD and AD in the retrieved countries

| Country | ASD Prevalence (cases per 10k) | AD prevalence (cases per 10k) |
| --- | --- | --- |
| Afghanistan | 91.2 | 351.479 |
| Albania | 83.3 | 176.379 |
| Algeria | 89.4 | 306.697 |
| American Samoa | 82 | 552.464 |
| Andorra | 74.1 | 355.33 |
| Angola | 82.8 | 384.04 |
| Argentina | 81.4 | 360.389 |
| Armenia | 78.2 | 193.672 |
| Australia | 88.2 | 391.078 |
| Austria | 72.6 | 299.223 |
| Azerbaijan | 81.9 | 193.551 |
| Bahamas | 82.7 | 384.511 |
| Bahrain | 103.3 | 349.662 |
| Bangladesh | 85.3 | 291.375 |
| Barbados | 81.1 | 377.209 |
| Belarus | 77.7 | 282.412 |
| Belgium | 73 | 393.375 |
| Belize | 85.9 | 383.931 |
| Benin | 81.9 | 294.972 |
| Bermuda | 85.4 | 384.222 |
| Bhutan | 88.4 | 290.672 |
| Bolivia | 83.9 | 500.026 |
| Bosnia and Herzegovina | 82.8 | 228.936 |
| Botswana | 84.1 | 447.279 |
| Brazil | 82.5 | 266.444 |
| Bulgaria | 80 | 208.058 |
| Cambodia | 80.1 | 334.283 |
| Cameroon | 82.5 | 304.418 |
| Canada | 86.4 | 541.588 |
| Central African Republic | 82.1 | 354.813 |
| Chad | 82.4 | 294.703 |
| Chile | 81.9 | 474.117 |
| China | 79.7 | 185.553 |
| Colombia | 85.8 | 341.521 |
| Comoros | 84 | 363.326 |
| Costa Rica | 87.7 | 327.241 |
| Croatia | 80.6 | 224.493 |
| Cuba | 83.3 | 383.353 |
| Cyprus | 73.9 | 300.662 |
| Czech Republic | 81.7 | 258.572 |
| Denmark | 73.8 | 579.908 |
| Djibouti | 81.5 | 361.154 |
| Dominica | 84.7 | 383.426 |
| Dominican Republic | 85.3 | 383.745 |
| DR Congo | 82.5 | 354.753 |
| Ecuador | 84.5 | 446.636 |
| Egypt | 89.4 | 218.713 |
| El Salvador | 83.6 | 297.128 |
| Equatorial Guinea | 83.3 | 352.352 |
| Eritrea | 83.8 | 363.556 |
| Estonia | 77.6 | 384.338 |
| Eswatini | 84.8 | 447.47 |
| Ethiopia | 83.4 | 353.798 |
| Fiji | 81.1 | 305.114 |
| Finland | 73.2 | 600.077 |
| France | 69.3 | 541.66 |
| Gabon | 83 | 355.427 |
| Gambia | 82.4 | 294.94 |
| Georgia | 78.9 | 177.97 |
| Germany | 72.2 | 385.066 |
| Ghana | 82 | 170.931 |
| Greece | 72.4 | 253.641 |
| Greenland | 85.5 | 494.872 |
| Grenada | 85.6 | 383.147 |
| Guam | 80.5 | 304.774 |
| Guatemala | 86.5 | 359.507 |
| Guinea | 82 | 295.07 |
| Guinea-Bissau | 81.3 | 295.082 |
| Guyana | 84.8 | 384.125 |
| Haiti | 83.7 | 384.472 |
| Honduras | 88 | 359.442 |
| Hungary | 79.9 | 317.761 |
| Iceland | 71.9 | 629.892 |
| India | 88.5 | 180.888 |
| Indonesia | 83.6 | 209.864 |
| Iran | 90.3 | 229.686 |
| Iraq | 89.4 | 249.222 |
| Ireland | 77.8 | 498.635 |
| Israel | 75.1 | 418.112 |
| Italy | 72 | 386.136 |
| Ivory Coast | 83.1 | 293.913 |
| Jamaica | 85 | 383.891 |
| Japan | 85.8 | 498.205 |
| Jordan | 92.1 | 307.049 |
| Kazakhstan | 80.7 | 184.12 |
| Kenya | 86.5 | 387.243 |
| Kiribati | 80.1 | 305.647 |
| Kuwait | 97.7 | 319.428 |
| Kyrgyzstan | 83.2 | 195.276 |
| Laos | 80.8 | 334.007 |
| Latvia | 76.3 | 266.637 |
| Lebanon | 89.8 | 380.144 |
| Lesotho | 84.8 | 447.824 |
| Liberia | 82.4 | 294.469 |
| Libya | 90.6 | 351.291 |
| Lithuania | 76.9 | 147.773 |
| Luxembourg | 74.6 | 552.517 |
| Madagascar | 82.8 | 363.214 |
| Malawi | 83.5 | 364.088 |
| Malaysia | 81.6 | 374.095 |
| Maldives | 82.6 | 330.923 |
| Mali | 82.7 | 259.918 |
| Malta | 74.3 | 322.657 |
| Marshall Islands | 82.2 | 305.075 |
| Mauritania | 83.3 | 294.862 |
| Mauritius | 79.8 | 334.198 |
| Mexico | 89.9 | 237.915 |
| Moldova | 80.3 | 282.327 |
| Mongolia | 82.3 | 194.14 |
| Montenegro | 83.2 | 231.336 |
| Morocco | 88.4 | 343.509 |
| Mozambique | 82.2 | 307.058 |
| Myanmar | 79.3 | 334.653 |
| Namibia | 82.9 | 447.974 |
| Nepal | 83.3 | 291.618 |
| Netherlands | 73.8 | 530.453 |
| New Zealand | 85.5 | 530.153 |
| Nicaragua | 87.5 | 359.131 |
| Niger | 81.9 | 294.767 |
| Nigeria | 82.9 | 275.436 |
| North Korea | 76.4 | 207.408 |
| North Macedonia | 83.5 | 214.115 |
| Northern Mariana Islands | 81.9 | 304.985 |
| Norway | 72 | 578.525 |
| Oman | 107.2 | 273.197 |
| Pakistan | 86.7 | 324.792 |
| Palestine | 91 | 322.728 |
| Panama | 87.4 | 447.072 |
| Papua New Guinea | 82.2 | 304.51 |
| Paraguay | 83.9 | 367.61 |
| Peru | 84.5 | 525.635 |
| Philippines | 81.8 | 374.017 |
| Poland | 81.2 | 304.457 |
| Portugal | 70.5 | 359.983 |
| Puerto Rico | 81.9 | 383.865 |
| Qatar | 151.2 | 346.123 |
| Republic of the Congo | 83.1 | 354.825 |
| Romania | 80 | 249.217 |
| Russia | 77.1 | 252.813 |
| Saint Lucia | 83.8 | 383.783 |
| Saint Vincent and the Grenadines | 84.8 | 383.51 |
| Samoa | 82.9 | 304.938 |
| Sao Tome and Principe | 82.6 | 294.76 |
| Saudi Arabia | 100.7 | 349.325 |
| Senegal | 81.6 | 294.405 |
| Serbia | 81.6 | 227.342 |
| Seychelles | 80.9 | 333.122 |
| Sierra Leone | 81.3 | 294.873 |
| Slovakia | 82 | 258.67 |
| Slovenia | 81.8 | 224.258 |
| Solomon Islands | 82.6 | 304.808 |
| Somalia | 83.9 | 362.639 |
| South Africa | 83.6 | 435.522 |
| South Sudan | 82.3 | 362.361 |
| Spain | 73 | 275.003 |
| Sri Lanka | 78.7 | 333.776 |
| Sudan | 90.1 | 288.655 |
| Suriname | 84.6 | 383.707 |
| Sweden | 90.8 | 743.77 |
| Switzerland | 73.8 | 552.498 |
| Syria | 91.9 | 211.235 |
| Taiwan | 77.2 | 227.94 |
| Tajikistan | 84.8 | 193.849 |
| Tanzania | 83.1 | 311.966 |
| Thailand | 78.1 | 370.098 |
| Timor-Leste | 82.9 | 333.683 |
| Togo | 82.1 | 294.852 |
| Tonga | 81.7 | 305.583 |
| Trinidad and Tobago | 82.9 | 383.668 |
| Tunisia | 87.9 | 363.272 |
| Turkey | 87.8 | 286.277 |
| Turkmenistan | 82.7 | 193.733 |
| Uganda | 84 | 363.941 |
| Ukraine | 77 | 253.448 |
| United Arab Emirates | 112.4 | 348.039 |
| United Kingdom | 78.1 | 647.23 |
| United States | 80.9 | 473.989 |
| United States Virgin Islands | 79.7 | 384.218 |
| Uruguay | 80.8 | 351 |
| Uzbekistan | 83 | 193.98 |
| Vanuatu | 81.5 | 304.945 |
| Venezuela | 87 | 373.105 |
| Vietnam | 80.5 | 279.215 |
| Yemen | 90.4 | 351.592 |
| Zambia | 84 | 363.653 |
| Zimbabwe | 84.5 | 448.154 |
